# Update on cpnDB: a reference database of chaperonin sequences

**DOI:** 10.1093/database/baz033

**Published:** 2019-03-01

**Authors:** Sarah J Vancuren, Janet E Hill

**Affiliations:** Department of Veterinary Microbiology, University of Saskatchewan, 52 Campus Drive, Saskatoon SK, Canada

## Abstract

cpnDB was established in 2004 to provide a manually curated database of type I (60 kDa chaperonin, CPN60, also known as GroEL or HSP60) and type II (CCT, TRiC, thermosome) chaperonin sequences and to support chaperonin sequence-based applications including microbial species identification, detection and quantification, phylogenetic investigations and microbial community profiling. Since its establishment, cpnDB has grown to over 25 000 sequence records including over 4 000 records from bacterial type strains. The updated cpnDB webpage (www.cpndb.ca) provides tools for text- or sequence-based searches and links to protocols, and selected reference data sets are available for download. Here we present an updated description of the contents and taxonomic coverage of cpnDB and an analysis of *cpn*60 sequence diversity.

## Introduction

Chaperonins are a diverse group of molecular chaperones found in virtually all prokaryotes and eukaryotes (1). The type I chaperonins (60 kDa chaperonin, CPN60, also known as GroEL or HSP60) are found in bacteria and a few archaea and in the mitochondria and chloroplasts of eukaryotes. Type II chaperonins include the eukaryote cytoplasmic CCT (for chaperonin containing TCP1, also called TRiC for TCP1 ring complex) and the archaeal homolog, thermosome. Their conservation across all domains of life makes the genes encoding chaperonin proteins attractive targets for use in phylogenetic investigations.

The discovery that a 549–567 bp region of the cpn60 gene termed the ‘universal target’ (UT; corresponding to nucleotides 271–825 of the *Escherichia coli* cpn60 gene) could be polymerase chain reaction (PCR) amplified with degenerate primers provided the foundation for development of numerous applications exploiting cpn60 sequences (2). Since that time, cpn60 UT sequences have been used as evidence for definition of new bacterial species (3, 4), provided targets for hybridization, PCR and sequencing-based diagnostics for bacteria (5–11) and for amplicon-based profiling of complex microbial communities (12–16). In addition, cpn60 UT sequences have proven to be excellent indicators of whole genome sequence similarities among bacteria (17–19). These applications are made possible by the sequence diversity of the UT region, especially among closely related taxa. Using criteria established by the International Barcode of Life project (20), the cpn60 UT has been demonstrated to be a preferred barcode for bacteria, compared to the 16S rRNA gene (21). The relatively large ‘barcode gap’ between inter- and intra-specific distances facilitates resolution of taxa, often at subspecies levels (10, 22–24). More recently, ‘universal’ primers for amplification of partial thermosome gene sequences were developed for archaea. As with the bacterial type I chaperonin, evidence to date suggests that these type II chaperonin sequences provide higher resolution than 16S rRNA gene sequences (25).

The development of chaperonin sequence-based methods inspired the original development of a reference database of chaperonin sequences. cpnDB was released in 2004 (26) and has been continuously maintained and updated since then. The database has been hosted at the University of Saskatchewan (Saskatoon, Canada) since 2015. cpnDB was designed to provide users with the ability to query records by sequence similarity search, or based on keywords, and to allow users to download sequences of interest for offline analyses. At its inception, cpnDB contained approximately 2000 type I and archaeal type II chaperonin sequence records representing 240 genera and has now grown to more than 25000 records (including eukaryotic type II chaperonin sequences) representing more than 1800 genera. cpnDB records are manually curated to ensure high quality entries. The original goal was to include all published type I and II chaperonin sequences, but since whole genome sequencing became a common activity and the volume of new sequence data increased exponentially, the emphasis has shifted to providing high quality records with broad taxonomic coverage, and with a particular emphasis on inclusion of type strains since they are the most useful landmarks for microbial species identification. A complete list of citations of cpnDB is maintained on the database webpage (http://www.cpndb.ca/publications.php).

The purpose of this update is to describe changes to the form and content of cpnDB since its original publication (26) and to provide a description of the taxonomic and sequence diversity represented in the database.

## Database access, structure and web interface

cpnDB is freely available to users through the web interface (http://www.cpndb.ca). There is no requirement for creation of an account, and no password is needed to access the database.

**Figure 1:**
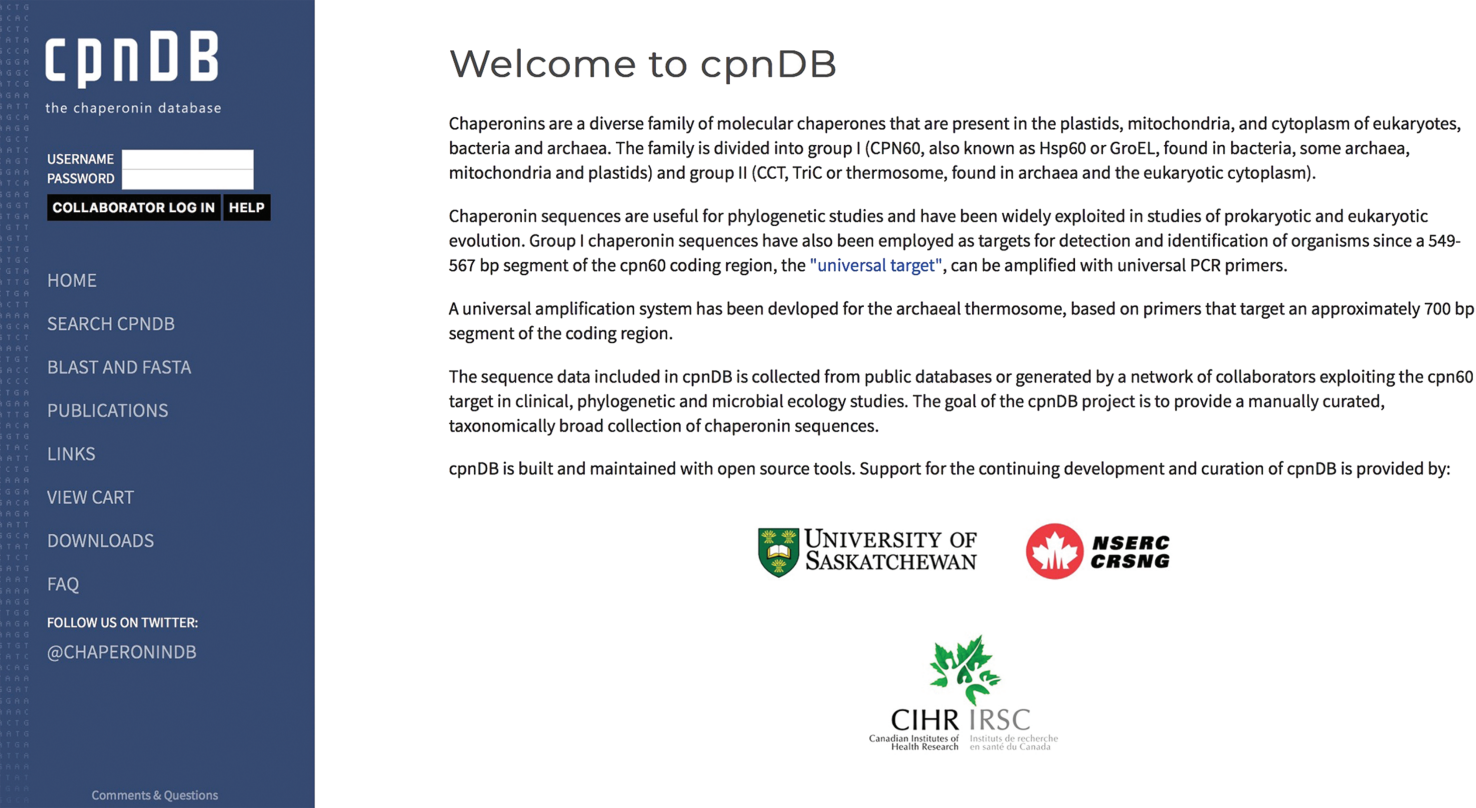
Homepage of cpnDB.

cpnDB was constructed with MySQL and the web interface is implemented with PHP. The web interface of cpnDB supports searching by text using terms such as genus and species names, culture collection catalog numbers or hierarchical taxonomy terms from the National Center for Biotechnology Information (NCBI) Taxonomy database (https://www.ncbi.nlm.nih.gov/guide/taxonomy/). Sequence searches can be done using BLASTP, primer blast (27) or FASTA (28). Users can choose to limit their search to type I (UT or full-length sequences) or type II chaperonin sequences. Individual records or groups of records for download can be selected from search results. The homepage provides information about chaperonins and links to publications citing cpnDB to provide information about applications of chaperonin sequences (Figure 1).

**Figure 2:**
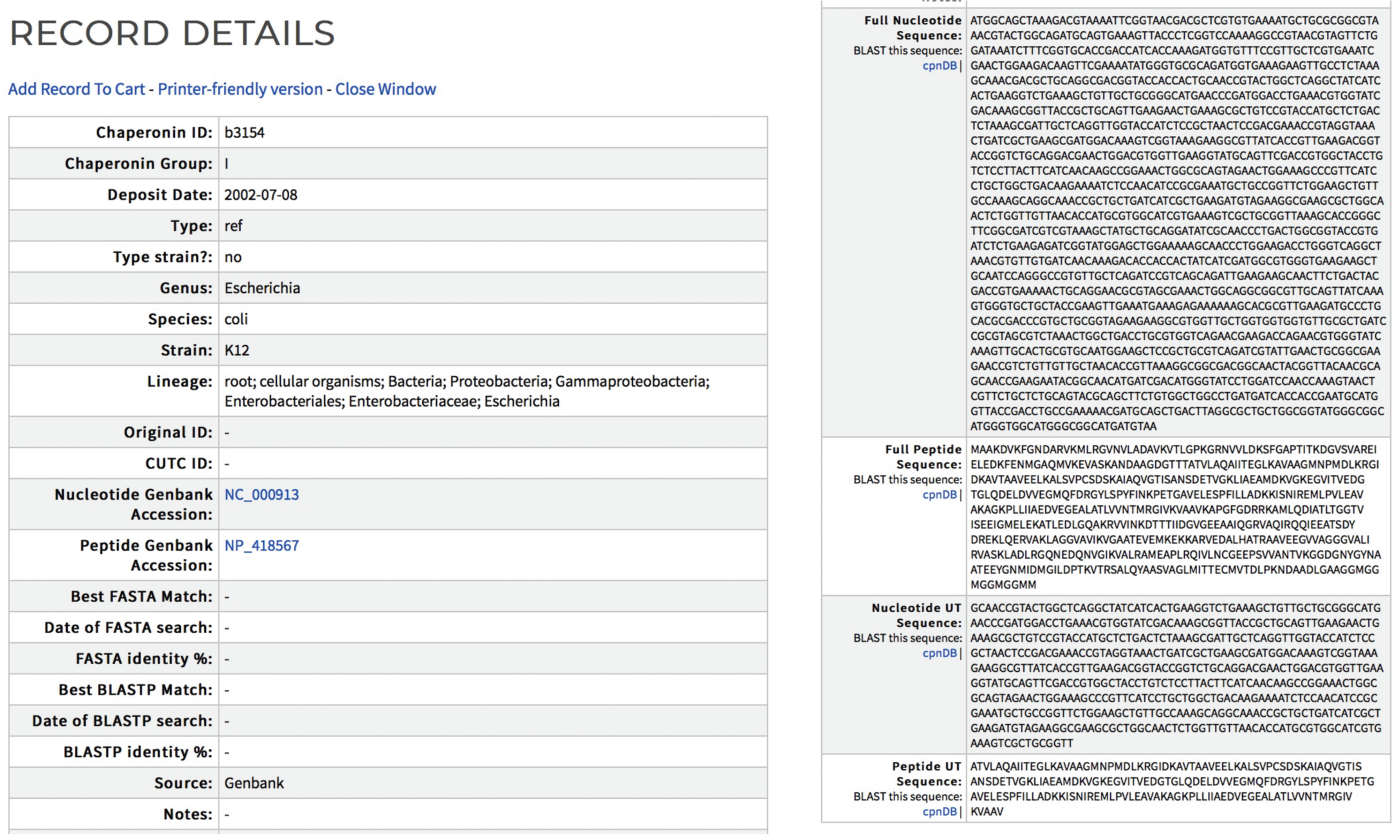
Sample cpnDB record for *E. coli* strain K12. External links to the corresponding Genbank record are included.

cpnDB records are assigned a unique identifier (Chaperonin ID). Each record contains the full-length chaperonin gene and protein sequences when available and the corresponding UT regions for type I sequences (Figure 2). Records also contain the taxonomic lineage of the source organism based on the NCBI Taxonomy ID. Strain name synonyms are provided for bacterial type strain records, using information extracted from the List of Prokaryotic Names with Standing in Nomenclature (http://www.bacterio.net) or relevant culture collection catalogs. Nucleotide and peptide Genbank accession numbers are provided, with external links. cpnDB is a LinkOut provider (https://www.ncbi.nlm.nih.gov/projects/linkout/), and so reciprocal links from Genbank records to the corresponding entry in cpnDB can be accessed from relevant Genbank records. cpnDB records for sequences generated from microbial community studies or clinical specimens include information about their closest match in the reference database (updated with each new addition).

**Table 1:** Taxonomic categories represented by Type I and II chaperonin sequences in cpnDB

Domain; (phylum, group or superphylum) ^a^	Number of Genera	Number of species
Type I		
Archaea; Euryarchaeota	10	13
Bacteria; Acidobacteria	7	9
Bacteria; Actinobacteria	162	725
Bacteria; Aquificae	8	13
Bacteria; Armatimonadetes	1	1
Bacteria; FCB group	139	379
Bacteria; PVC group	32	51
Bacteria; Chloroflexi	12	15
Bacteria; Chrysiogenetes	1	1
Bacteria; Cyanobacteria	44	64
Bacteria; Deferribacteres	5	5
Bacteria; Deinococcus–Thermus	6	23
Bacteria; Dictyoglomi	1	2
Bacteria; Elusimicrobia	1	1
Bacteria; Firmicutes; Clostridia	107	286
Bacteria; Firmicutes; Bacilli	92	545
Bacteria; Firmicutes; Mollicutes	4	17
Bacteria; Firmicutes; Erysipelotrichia	9	10
Bacteria; Fusobacteria	7	20
Bacteria; Gemmatimonadetes	1	1
Bacteria; Nitrospirae	3	4
Bacteria; Proteobacteria; Alphaproteobacteria	194	491
Bacteria; Proteobacteria; Betaproteobacteria	89	233
Bacteria; Proteobacteria; Gammaproteobacteria	214	764
Bacteria; Proteobacteria; Delta/Epsilon subdivisions	63	151
Bacteria; Spirochaetes	11	78
Bacteria; Synergistetes	8	9
Bacteria; Tenericutes	5	23
Bacteria; Thermodesulfobacteria	2	3
Bacteria; Thermotogae	8	15
Eukaryota; Archaeplastida	67	79
Eukaryota; SAR supergroup	32	50
Eukaryota; Excavata	9	20
Eukaryota; Amoebozoa	4	10
Eukaryota; Opisthokonta; Metazoa	83	94
Eukaryota; Opisthokonta; Fungi	57	132
Type II		
Archaea; Euryarchaeota	66	158
Archaea; DPANN	1	1
Archaea; Proteoarchaeota	27	46
Eukaryota; Archaeplastida	25	28
Eukaryota; SAR supergroup	21	46
Eukaryota; Excavata	5	12
Eukaryota; Amoebozoa	5	8
Eukaryota; Opisthokonta; Metazoa	89	106
Eukaryota; Opisthokonta; Fungi	53	77
Eukaryota; Hacrobia	4	4

^a^Based on NCBI Taxonomy

**Figure 3:**
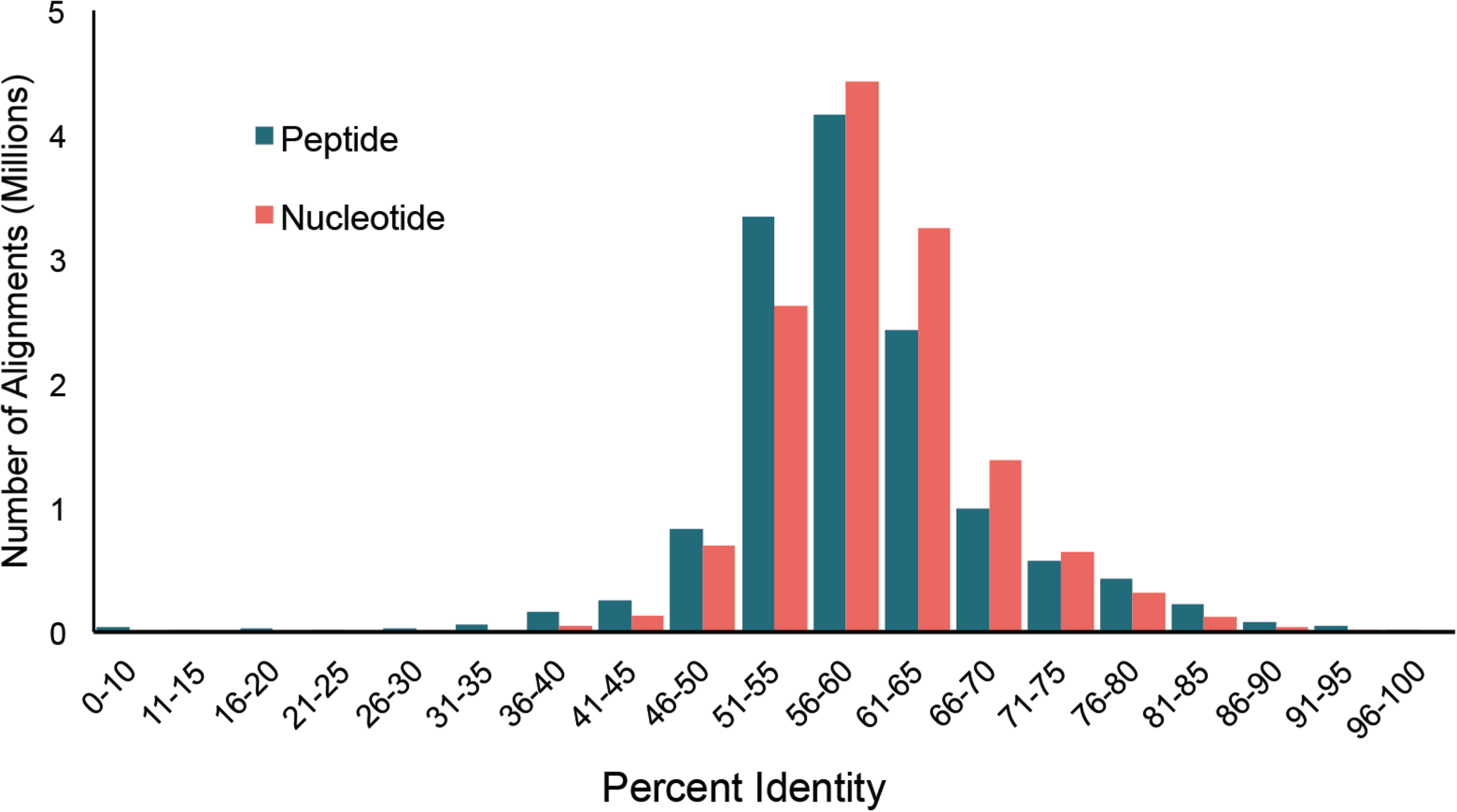
Distribution of pairwise percent identities for bacterial cpn60 UT sequences in cpnDB_nr (version 11 May 2018; 5235 species).

**Figure 4:**
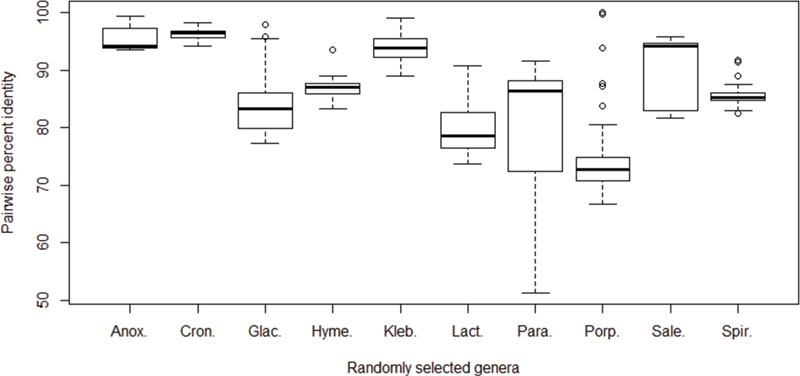
Pairwise percent identities of the *cpn*60 gene among species. The *cpn*60 sequences of species from 10 randomly selected bacterial genera with at least 6 unique species records were aligned to calculate pairwise identities. Genera from left to right: *Anoxybacillus* (6 species), *Cronobacter* (6 species), *Glaciecola* (10 species), *Hymenobacter* (6 species), *Klebsiella* (9 species), *Lactococcus* (7 species), *Paracoccus* (9 species), *Porphyromonas* (17 species), *Salegentibacter* (6 species) and *Spirosoma* (7 species).

BLAST and FASTA databases are updated with each addition of new records to cpnDB, and the entire sequence collection can be downloaded in FASTA format. A non-redundant version of the database, cpnDB_nr, is similarly updated with each new addition. This subset of the database is limited to type I chaperonins (bacteria, archaea and eukaryotes) and includes only a single representative sequence from each species, with priority given to the type strain when available. Sequence-based queries of cpnDB_nr may be desirable when a broad view of the relationship of the query to chaperonin sequences from other taxa is wanted, since results will include more different species rather than multiple strains of one or a few closely related species. cpnDB_nr can also be downloaded (nucleotide or peptide UT sequences) for use in offline analyses, and versions are identified on the download page by the creation date.

## Sources of data and curation methods

All records in cpnDB are manually curated to ensure they contain complete sequences with no ambiguous positions and are correctly annotated. UT sequences from full-length type I chaperonin (cpn60) sequences are identified based on location of sequence regions corresponding to the predicted annealing sites of the degenerate PCR primers that are used to amplify the UT region, H279 and H280 (2). Direct submissions to cpnDB are not accepted, but curators encourage users to deposit sequences to public databases, which are surveyed weekly with PubCrawler (29). Sequences from newly identified genera and species, especially bacterial type strains or reference strains with thorough annotation, are prioritized for inclusion.

## Database contents and scope

Since the release of cpnDB in 2004 (26), the database has grown from approximately 2000 records representing 247 genera to over 25 000 records representing 1802 genera, with a corresponding increase in taxonomic coverage (Table 1). Not surprisingly, since cpnDB records are primarily drawn from public databases, the highest numbers of entries are from organisms in the most well-studied bacterial phyla, with 1070/1802 genera belonging to phyla Firmicutes, Actinobacteria, Proteobacteria and Bacteroidetes. The number of species per genus ranges from 1 to over 200 (*Streptomyces* spp.).

Bacterial type I chaperonin (cpn60) sequences are the most widely exploited for research and diagnostics. To investigate sequence diversity among bacterial type I chaperonin sequences in cpnDB, pairwise percent identities were calculated for the type I bacterial sequences from cpnDB_nr (version 25 November 2018) nucleotide and peptide UT sequences (5235 sequences for each) using Clustalw2 (30). In both cases, normal distributions of pairwise percent identity values were observed, with median values of 59.8% and 58.1% for nucleotide and peptide sequences, respectively (Figure 3). The pattern is strikingly similar to the distribution of nucleotide and peptide UT sequence identities calculated for representatives of the original 247 genera included in cpnDB (26), indicating that while the depth and scope of cpnDB have increased, the spectrum of chaperonin sequence diversity has not expanded substantially beyond what was initially described in 2004.

The 16S rRNA gene sequences are commonly used markers for describing bacterial diversity and an ~98.7–99% identity recommended for demarcation of species based on 16S rRNA sequences (31). More rapidly evolving protein-coding genes offer superior resolution (17, 32, 33), which supports their exploitation in diagnostics since lower sequence identities between closely related taxa makes their discrimination more obvious and technically tractable. To investigate inter-species cpn60 UT sequence identities, we randomly selected the following 10 bacterial genera for which there were at least 6 species available in cpnDB: *Anoxybacillus* (6 species), *Cronobacter* (6 species), *Glaciecola* (10 species), *Hymenobacter* (6 species), *Klebsiella* (9 species), *Lactococcus* (7 species), *Paracoccus* (9 species), *Porphyromonas* (17 species), *Salegentibacter* (6 species) and *Spirosoma* (7 species). cpn60 UT nucleotide sequences for each genus were aligned and pairwise identities were calculated (Figure 4). Median identities were highly variable among genera, ranging from 72.8% for *Porphyromonas* to 96.4% for *Cronobacter*. There was also a wide of range of values observed within genera, and in three cases (*Glaciecola*, *Paracoccus* and *Porphyromonas*), the range exceeded 20%. This wide distribution of identities within genera is consistent with previously published observations of *Campylobacter* (71–92% pairwise identity among 15 species; 23), *Parabacteroides* (83–97% among 5 species; 34), *Prevotella* (68–94% among 38 species; 34) and *Enterococcus* (78%–88% among 18 species; 9). A previous study comparing cpn60 UT and 16S rRNA gene sequence diversity within a set of 983 bacterial species from 21 phyla similarly concluded that cpn60 UT intra- and inter-specific identities were more broadly distributed and almost always lower than corresponding 16S rRNA gene sequence identities (21).

## Conclusions and future directions

cpnDB has supported chaperonin sequence-based research and diagnostics since 2004 and will continue to be updated as new genomes are sequenced and species are discovered to ensure its continuation as a source for reference sequences of chaperonin genes representing the broadest possible taxonomic range. Curation will continue to focus on reference sequence data from microbial taxa, but additional complementary resources focused on curation and analysis of data generated in microbiome studies using cpn60 amplicon sequencing are also being developed.

cpnDB remains one of very few sources of sequence barcode information, along with the Ribosomal Database Project (ribosomal RNA encoding genes for bacteria, archaea and fungi; 35) and the Barcode of Life Data System (cytochrome oxidase I genes for plants, animals, protists and fungi; 36). The accessibility of chaperonin UT sequences with degenerate primer PCR coupled with the superior resolution of these usually single-copy sequences will continue to make chaperonin sequences attractive barcodes for detection, identification and quantification of organisms in isolation or in complex microbial communities.

## Notes

**Database URL:** http://www.cpndb.ca/
